# Using corneal confocal microscopy to compare Mecobalamin intramuscular injections vs oral tablets in treating diabetic peripheral neuropathy: a RCT

**DOI:** 10.1038/s41598-021-94284-4

**Published:** 2021-07-19

**Authors:** Yuanjin Zhang, Dongsheng Fan, Yixuan Zhang, Shuo Zhang, Haikun Wang, Ziyuan Liu, Hongli Wang

**Affiliations:** 1 Neurology Department, Peking University Third Hospital, 49 North Garden Rd., Haidian District, Beijing, 100191 China; 2 Beijing Municipal Key Laboratory of Biomarker and Translational Research in Neurodegenerative Disease, Beijing, China; 3 Department of Ophthalmology, Peking University Third Hospital, Beijing, China

**Keywords:** Diseases, Endocrinology, Medical research, Neurology

## Abstract

This randomized controlled study used corneal confocal microscopy (CCM) to compare the efficacy of Mecobalamin intramuscular injections vs oral tablets in treating mild to moderate diabetic peripheral neuropathy (DPN) by detecting early nerve fiber repair. Enrolled patients were randomized approximately 1:1 to receive Mecobalamin intramuscular injections (0.5 mg/day, 3 times/week) or Mecobalamin oral tablets (1.5 mg/day) for 8 weeks. Primary outcome was change of inferior whorl length (IWL) from baseline. Secondary outcomes included changes of corneal nerve fibre length (CNFL), corneal nerve fibre density (CNFD), corneal nerve branch density (CNBD) and the Survey of Autonomic Symptoms (SAS). 15 (93.75%) patients in the injection group and 17 (89.47%) patients in the tablet group completed the study. The injection treatment significantly improved patients’ IWL from baseline (21.64 ± 3.00 mm/mm^2^ vs 17.64 ± 4.83 mm/mm^2^, *P* < 0.01) while the tablet treatment didn’t. Additionally, the injection treatment led to significantly improved CNFL, CNBD and SAS from baseline (all *P* < 0.05) while the tablet treatment did not. No patient experienced any adverse events. In conclusion, CCM is sensitive enough to detect the superior efficacy of 8-week Mecobalamin intramuscular injection treatment for DPN compared to the oral tablet treatment.

ClinicalTrials.gov registration number: NCT04372316 (30/04/2020).

## Introduction

Diabetic peripheral neuropathy (DPN) is a common chronic complication of Types 1 and 2 diabetes that affects up to 50% patients with diabetes^1–3^. Both somatic and autonomic components of the nerve system are involved^1,3^. DPN is defined as a “symmetrical, length-dependent sensorimotor polyneuropathy attributable to metabolic and microvessel alterations resulting from chronic hyperglycemia and cardiovascular risk covariates” by the Toronto Consensus Panel on diabetic neuropathy^3^. It is often characterized by severe neuropathic pain, sensory loss and paresthesia, and could lead to foot ulceration, infection and ultimately lower-limb amputation^1–4^. Early presentations of DPN are usually caused by reversible biochemical and functional peripheral nerve changes, and these early DPN presentations are often missed. When a diagnosis of DPN is finally made in a patient, the underlying peripheral nerve changes have often progressed to become irreversible structural damage^1,4^. Therefore, early detection of incipient peripheral nerve changes is important. There have only been a few symptomatic and palliative treatments for DPN, and currently there is no Food and Drug Administration (FDA) approved treatment for modifying DPN’s underlying pathophysiological changes^5,6^. The lack of disease modifying treatment for DPN could be due to inappropriate treatment targets, advanced DPN stage and/or lack of adequate test to properly assess nerve fibre repair that is reflective of therapeutic benefits^5,7^.

Nerve conduction studies (NCS) are the gold standard for DPN diagnosis and evaluation, however, a NCS only assesses large nerve fibre functions, it does not assess small fibre functions such as functions of thinly myelinated Aδ fibre and unmyelinated C fibres, both of which are heavily involved in DPN^1,5^. Small fibres are damaged early in DPN development^1,5^. Currently, intra-epidermal nerve fibre density (IENFD) in skin biopsy is considered to be the best measure for assessing small nerve fibre damage, however, it is invasive, complex and has low patient acceptance and limited use in assessing benefits of DPN treatments^8^.

In vivo corneal confocal microscopy (CCM) is a rapid, easy, non-invasive, reproducible and repeatable ophthalmic imaging technique that enables visualization of Aδ and unmyelinated C fibres in the heavily innervated cornea^5,7–10^. Damage of Aδ and unmyelinated C fibres causes most of the DPN symptoms^9^. It has been demonstrated that CCM could quantitatively assess small fibre injury and has become a validated tool for diagnosing DPN in both types 1 and 2 diabetes^7–10^. CCM could detect early DPN and its assessment of corneal nerve damage could be associated with DPN severity^5,7–10^. CCM assessments of nerve fibre damage had similar sensitivity and specificity to those of IENFD^10^. Most importantly, CCM’s utility as a surrogate endpoint for assessing benefits of DPN treatments has been demonstrated by its ability to detect early nerve fibre repair by detecting corneal nerve branch density (CNBD) increase followed by increases in corneal nerve fibre length (CNFL) and density (CNFD) after simultaneous pancreas and kidney transplantation (SPK) for patients with Type 1 diabetes^7^. It has also been reported that CCM could detect improvement of corneal nerve morphology reflected by improved CNFD and corneal nerve fibre tortuosity upon improvement of DPN risk factors^11^. Several additional studies also supported the position that CCM allows for a precise and reliable quantification of both small nerve fiber damage and regeneration^12–22^, and recently it has been suggested that CCM could be included as a primary endpoint in clinical studies of DPN disease-modifying treatments^23^. Nevertheless, it is obvious that more studies are needed to further evaluate the feasibility of using CCM parameters as surrogate efficacy endpoints for nerve fibre repair in DPN treatments.

Mecobalamin is one of the two activated coenzyme forms of vitamin B12, it could enhance neuronal methylation, accelerate nerve cell growth and reduce homocysteine level, and therefore, it has neuroprotective effects^4^. Numerous studies have reported that intravenous, intramuscular or oral Mecobalamin treatment alone or in combination with other agents could promote peripheral nerve regeneration and improve clinical symptoms such as neuropathic pain as well as neurophysiological parameters such as nerve conduction velocity (NCV) in patients with DPN, although the magnitude of its benefits remains controversial^4,24–28^. Mecobalamin has been approved by the China Food and Drug Administration (CFDA) for treating peripheral neuropathy, and is recommended in the Chinese guideline for treating type 2 diabetes^28^.

There has been no study comparing the efficacy and safety of Mecobalamin intramuscular injections with Mecobalamin oral tablets in treating patients with DPN using CCM. In the current study, we compared efficacy and safety of Mecobalamin intramuscular injections vs Mecobalamin oral tablets in treating patients with mild to moderate DPN using CCM, such a study could evaluate the utility of CCM parameters as surrogate efficacy endpoints in DPN treatments as well as determine the more effective route of administration for Mecobalamin in treating patients with DPN.

## Methods

### Overall study design

This was a 8-week, single center, prospective, open-label, assessor-blind, case-controlled clinical trial conducted at Peking University Third Hospital China. This study was approved by the Medical Science Research Ethics Committee of the Peking University Third Hospital (Approval number: 286-02) and was conducted in accordance with Good Clinical Practice guidelines of the CFDA as well as the Declaration of Helsinki. Its *ClinicalTrials.gov* registration number is NCT04372316 (30/04/2020). All patients gave written informed consent before screening.

The study was originally designed to consist of a 8-week period during which patients were randomized approximately 1:1 to receive either Mecobalamin intramuscular injections (0.5 mg/day, 3 times/week) or Mecobalamin oral tablets (0.5 mg/time, 3 times/day) for 8 weeks, and a subsequent 24-week period during which all of the enrolled patients received Mecobalamin oral tablets (0.5 mg/time, 3 times/day). However, due to the COVID-19 pandemic, after the 8-week treatment period and data collection, it became very difficult for some patients to come back for follow-up tests. Therefore, only results from the 8-week treatment are reported.

Eligible patients visited our hospital (Peking University Third Hospital) for screening (baseline visit). Enrolled patients were asked to visit for randomization and at the end of the 8-week treatment. At baseline, the following data were collected: demographic information such as age, routine blood work, duration of diabetes, glycated hemoglobin (HbA1c) level, fasting blood glucose (FBG), the Toronto Clinical Neuropathy Score (TCSS), the Survey of Autonomic symptoms Scale (SAS), sympathetic skin response (SSR), sural nerve amplitude potential (SNAP), and CCM parameters including inferior whorl length (IWL), corneal nerve fibre length (CNFL), corneal nerve fibre density (CNFD) and corneal nerve branch density (CNBD).

### Patients

Male or female patients with mild (TCSS 6–8) to moderate (TCSS 9–11) DPN aged 18–70 years old who had been diagnosed with type 2 diabetes for at least 1 year with optimally controlled blood glucose level (HbA1c ≤ 9%) and who visited the Department of Neurology and Endocrinology, Peking University Third Hospital from December 2018 to August 2020 were screened. DPN was defined according to the Toronto Diabetic Neuropathy Expert Group recommendation^29^. Inclusion criteria: (1) Had distal, symmetrical, sensorimotor multiple peripheral neuropathy according to neuro-electrophysiological examinations, (2) no history of eye trauma, keratopathy, other intraocular ophthalmic disease(s) or corneal laser treatment, (3) not wearing contact lenses, (4) had not taken medications affecting corneal metabolism, (5) did not receive Mecobalamin or α-lipoic acid therapy within 3 months prior to the screening, (6) women of child bearing age with a negative urine pregnancy test who used effective contraception during and within 1 month after the treatment, (7) willing and able to comply with study visit schedule, treatment plan, laboratory tests and other study procedures.

Exclusion criteria: (1) Had been diagnosed with a malignant tumor within 2 years prior to the screening, (2) presence of other neurological disorders or skin lesions that researchers believed could potentially affect DPN evaluation, (3) fingertip amputation or tip of toe amputation, (4) participated in any other study involving a study drug or post-marketing drug within 30 days prior to the screening, (5) had clinically significant or unstable diseases such as but not limited to acute cardiovascular diseases, cerebrovascular diseases, liver diseases, kidney diseases, respiratory diseases, hematological diseases, immune system diseases, inflammatory or rheumatic diseases, uncontrolled infections, symptomatic peripheral vascular diseases, or untreated endocrine diseases, (6) had donated blood within 30 days prior to treatment initiation or planned to donate blood during the study period or within 30 days after the study, (7) white blood cells count (WBC) < 4000/mm^3^, neutrophils count < 1500/mm^3^, or platelet count < 10,00/mm^3^, (8) clinically significant abnormalities in 12- lead electrocardiogram (ECG), (9) received combined transcutaneous electrical nerve stimulation (TENS) or acupuncture, (10) a history of intolerance or allergy to Mecobalamin or similar chemical compound, or current presence or a history of alcohol and/or other substance abuse within 1 year prior to the screening, (11) other severe acute, chronic medical or psychiatric conditions or laboratory abnormalities that, as determined by the investigators, could potentially increase the risk associated with Mecobalamin treatment, affect interpretation of the study results, or render the subject unfit for the trial, (12) had vitamin B12 deficiency (serum vitamin B12 < 148 pmol/L), or (13) inability and/or unwillingness to understand and/or comply with the treatment plan.

### Randomization and treatment

Enrolled patients were randomized approximately 1:1 to receive Mecobalamin injections or Mecobalamin oral tablets by random number table. Unique identification number was established by investigators for each participating patient after the screening. Randomization and treatment initiation took place within 4 weeks after the screening.

Patients randomized to receive intramuscular Mecobalamin (methycobal) injections (Eisai China, Shanghai, China) received one 0.5 mg injection 3 times/week (administered every other day each week) for 8 weeks. Patients randomized to receive oral Mecobalamin tablets (Eisai China, Shanghai, China) took one 0.5 mg tablet 3 times/day for 8 weeks. As the 2 treatments had different routes of administration, the study was open-label. During the study, participants took antidiabetic drugs to maintain their blood glucose level as stable as possible. Medications treating the patients’ other diseases not prohibited in the inclusion and exclusion criteria above were allowed. Use of neurotrophin, nerve growth factor, epalrestat or other drugs for treating DPN was prohibited.

### Corneal confocal microscopy (CCM)

At the screening (baseline) and the end of the 8-week treatment, both eyes of each enrolled patient were examined by two qualified, treatment-blinded optometrists using laser corneal confocal microscope (Heidelberg Retinal Tomograph III Rostock Cornea Module, Heidelberg, Germany) to capture CCM images of the central and inferior whorl (IW) area of their cornea^7,30–32^. Three images from the central cornea and three from the IW area at the level of sub-basal nerve plexus were selected from each eye based on their quality and variability and quantified by semiautomatic Java-based image processing software (ImageJ, National Institutes of Health, Bethesda, MD, USA), and the NeuronJ image plug-in (Biomedical Imaging Group, Lausanne, Switzerland) was used to facilitate tracing and quantification. 4 corneal nerve parameters were quantified: (1) inferior whorl length (IWL = total length of nerves [mm/mm^2^] at the IW region), (2) corneal nerve fibre length (CNFL = total length of main nerves and nerve branches [mm/mm^2^]), (3) corneal nerve fibre density (CNFD = total number of main nerves/mm^2^), and (4) corneal nerve branch density (CNBD = number of nerve branches/mm^2^)^7,30–32^. Analysis was performed by 2 independent investigators blinded to the patients’ identification numbers and their treatments.

### Electrophysiological testing

Patients’ SSR and SNAP were measured at baseline and at the end of the 8-week treatment. SSR was measured on both hands and both feet. For the hand, the active electrode was placed on the palm and the reference electrode was placed at the corresponding position on the dorsum. For the foot, the active electrode was placed on the sole and the reference electrode was placed at the corresponding position on the dorsum. The ground electrode was placed on the left wrist. Electric stimuli were delivered with a stimulating electrode placed on the skin covering the left medium nerve 3 cm proximal to the ground electrode (anode proximal, cathode distal). Electric stimuli were delivered with an intensity of 30 milliamps (mA) lasting 0.15 ms (ms). The sensitivity was set at 0.5 millivolts (mV) per division. Each stimulus could be repeated no more than 2 times with an interval ≥ 90 s in order to obtain the optimal waveform. Latency (SSR Lat) was defined as the interval from the stimulation onset to the first peak or trough, while amplitude (SSR Amp) was measured peak-to-trough.

For SNAP testing, each patient was asked to lie down on his/her side and bend his/her legs. The active recording electrode was positioned at the patient’s ankle just behind the lateral malleolus, and the reference electrode was place on the dorsum 2-3 cm from the recording electrode. The stimulating electrode was place 14 cm proximally slightly lateral to the midline in the posterior aspect of the leg, with its cathode close to the recording electrode. The ground electrode was positioned between the recording electrode and the stimulating electrode. Electric stimuli of supramaximal intensity were delivered at 1 Hz lasting 0.1 ms and at a sweep speed of 2 ms/cm.

### Outcome measures

Primary efficacy outcome was IWL change from baseline at the end of the 8-week treatment. Secondary CCM outcomes included CNFL, CNFD and CNBD changes from baseline at the end of the 8-week treatment, and secondary clinical outcomes included changes of the TCSS score^33–35^ and the SAS score^10,36^ from baseline at the end of the 8-week treatment evaluated and analyzed by 2 independent investigators blinded to the patients’ identification numbers and their treatments.

### Statistical analysis

The SPSS software (IBM, Armonk, NY, USA) was used for all statistical analyses in the study. Data were expressed as means ± standard deviations (SD) for continuous variables and as n (%) for categorical variables. Per protocol set (all participants receiving the treatment in compliance with the protocol) (PPS) was used for efficacy analysis. Paired *t* test was used to compare within-group changes of primary and secondary outcomes from baseline, *t* test or non-parametric test was used for inter-group comparisons. Pearson’s correlation coefficient (*r*) was used to assess relationships between significant change(s) of CCM parameters from baseline and significant change(s) of clinical outcomes from baseline. All tests were two-tailed (α = 0.05), and a *P* value < 0.05 indicated statistical significance.

## Results

### Demographics and baseline characteristics

**Figure 1:**
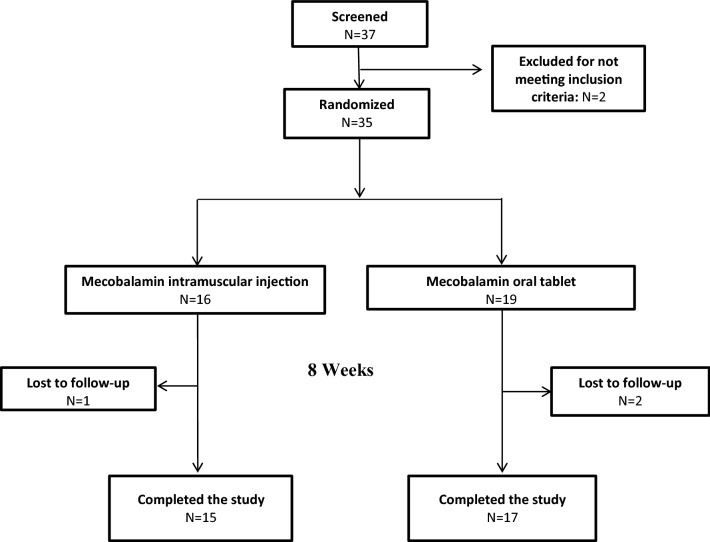
Study flow diagram.

Study flow diagram was illustrated in Fig. 1. 37 eligible patients were screened and 2 were excluded for not meeting the study inclusion criteria. 35 patients were enrolled in our study and randomized to receive Mecobalamin intramuscular injection treatment (N = 16) or oral Mecobalamin tablet treatment (N = 19) for 8 weeks. During the 8-week treatment, 1 patient in the Mecobalamin injection group and 2 patients in the Mecobalamin tablet group were lost to follow-up. In total, 15 (93.75%) patients in the Mecobalamin injection group and 17 (89.47%) patients in the Mecobalamin tablet group completed the 8 week-treatment in compliance with the treatment protocol and these patients constituted the PPS population. Dates defining the periods of recruitment and follow-up were December 2018 to August 2020 and July 2019 till now, respectively.

Demographics and baseline characteristics of the PPS population were listed in Table 1. Patients in the Mecobalamin injection group had significantly higher baseline TCSS and SAS scores and significantly shorter baseline IWL than patients in the Mecobalamin tablet group (Table 1), suggesting that patients in the Mecobalamin injection group could have more severe DPN^10,33,34,36^. Patients in the 2 treatment groups had comparable demographics and other baseline characteristics (Table 1).

**Table 1:** Patients demographics and baseline characteristics (PPS).

	Mecobalamin injection group (N = 15)	Oral Mecobalamin tablet group (N = 17)	*P* value
Age (year)	59.07 ± 9.98	56.59 ± 9.67	0.79
HbA1c (%)	7.51 ± 1.16	7.54 ± 1.79	0.96
Duration of diabetes (year)	12.97 ± 7.61	11.09 ± 7.83	0.83
FBG (mmol/L)	8.58 ± 2.30	8.52 ± 2.25	0.94
TCSS (points)	6.67 ± 2.69	4.29 ± 1.31	0.01*
SAS	4.53 ± 2.61	1.65 ± 1.27	< 0.01*
Left sural SNAP (µV)	6.79 ± 5.25	8.72 ± 5.14	0.22
Upper extremity SSR Lat (ms)	1391.20 ± 460.18	1499.47 ± 458.42	0.49
Upper extremity SSR Amp (mV)	1.43 ± 1.20	0.97 ± 0.75	0.14
Lower extremity SSR Lat (ms)	1655.40 ± 543.36	1772.76 ± 363.62	0.79
Lower extremity SSR Amp (mV)	0.46 ± 0.37	0.44 ± 0.33	0.77
IWL (mm/mm^2^)	17.64 ± 4.83	22.10 ± 4.74	0.01*
CNFL (mm/mm^2^)	17.60 ± 4.31	19.67 ± 3.43	0.14
CNFD (n/mm^2^)	32.64 ± 7.70	35.79 ± 8.32	0.40
CNBD (n/mm^2^)	40.28 ± 24.00	51.84 ± 20.10	0.17

Data are expressed as means ± standard deviations unless otherwise indicated.

Abbreviations: *PPS* per protocol set, *HbA1c* glycated hemoglobin, *FBG* fasting blood glucose, *TCSS* Toronto clinical neuropathy score, *SAS* survey of autonomic symptoms, *SNAP* sural nerve amplitude potential, *SSR* sympathetic skin response, *Lat* latency, *Amp* amplitude *IWL* inferior whorl length, *CNFL* corneal nerve fiber length, *CNFD* corneal nerve fiber density, *CNBD* corneal nerve branch density.

*P value < 0.05.

### Primary efficacy outcome

**Figure 2:**
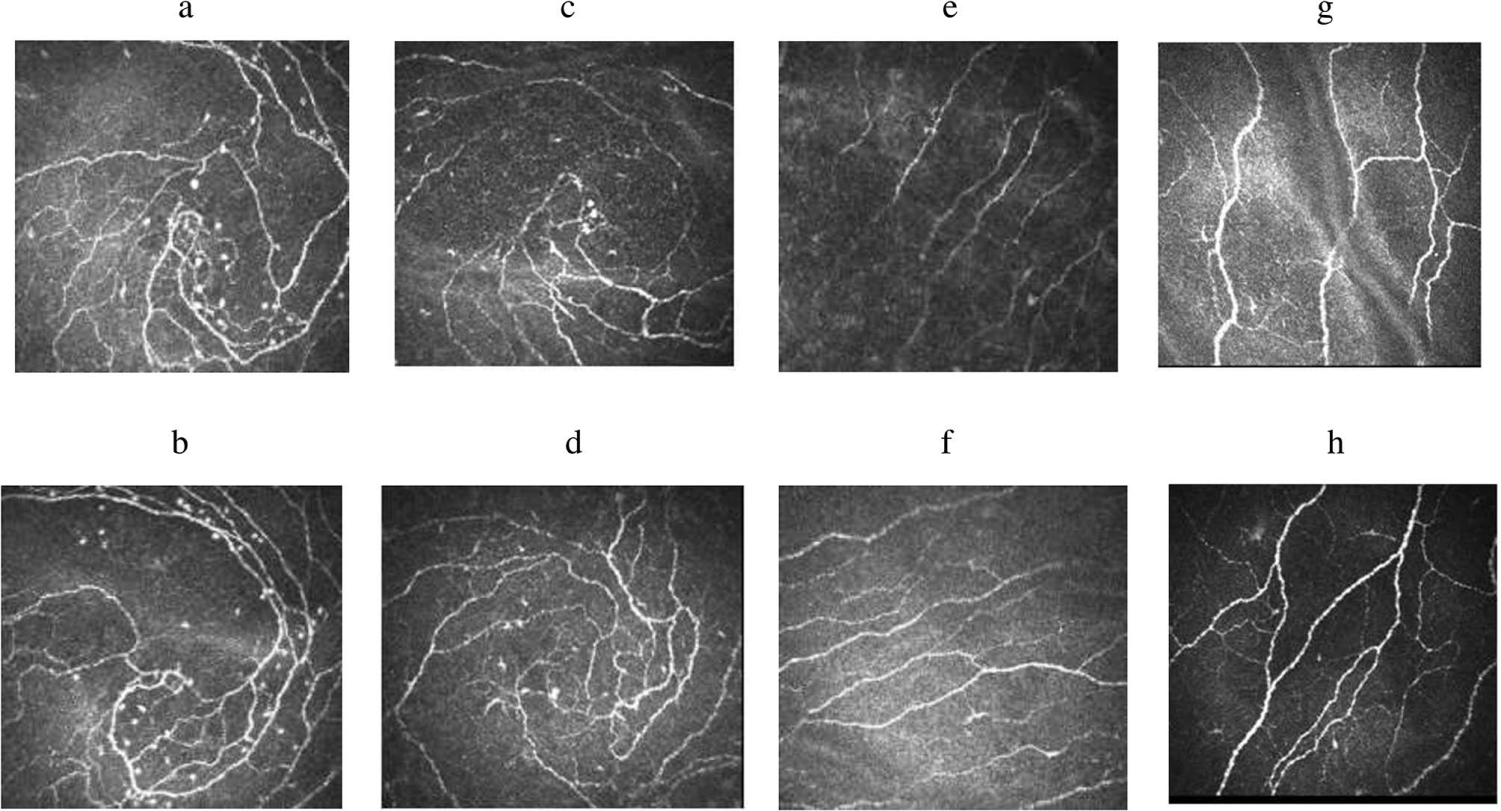
Corneal confocal microscopic images of morphological changes of the inferior whorl (IW) nerve complex pattern and the conventional sub-basal nerve plexus pattern around the central cornea after Mecobalamin treatment. (**a,b)** The IW nerve complex patterns before (**a**) and after (**b**) Mecobalamin intramuscular injection treatment. (**c,d)** The IW nerve complex conventional sub-basal nerve plexus pattern around the central cornea patterns before (**c**) and after (**d**) oral Mecobalamin tablet treatment. (**e,f)** The conventional sub-basal nerve plexus pattern around the central cornea patterns before (**e**) and after (**f**) Mecobalamin intramuscular injection treatment. (**g,h)** The conventional sub-basal nerve plexus pattern around the central cornea patterns before (**g**) and after (**h**) oral Mecobalamin tablet treatment.

Patients in the Mecobalamin injection group had significantly improved IWL at the end of the treatment compared to baseline (21.64 ± 3.00 mm/mm^2^ vs 17.64 ± 4.83 mm/mm^2^, *P* < 0.01) while patients in the tablet group did not (21.88 ± 4.27 mm/mm^2^ vs 22.10 ± 4.74 mm/mm^2^, *P* = 0.816). The Mecobalamin injection treatment also led to significantly greater IWL improvement than the Mecobalamin tablet treatment (4.00 ± 3.09 mm/mm^2^ vs -0.22 ± 3.92 mm/mm^2^, *P* = 0.002). Figure 2a–d were CCM images of the IW nerve complex pattern before and after the Mecobalamin treatment, from which a patient’s IWLs before and after the treatment were measured.

### Secondary efficacy outcomes

Patients in the Mecobalamin injection group had significantly greater CNFL improvement at the end of 8 weeks than patients in the Mecobalamin tablet group (3.26 ± 4.01 mm/mm^2^ vs 0.08 ± 4.64 mm/mm^2^, *P* = 0.048), while CNFD and CNBD improvement associated with the Mecobalamin injection treatment were numerically though insignificantly greater than the tablet treatment (both *P* > 0.05) (Table 2). The Mecobalamin injection treatment significantly improved patients’ CNFL (20.86 ± 3.46 mm/mm^2^ vs 17.60 ± 4.31 mm/mm^2^, *P* = 0.01) and CNBD (51.76 ± 16.00 n/mm^2^ vs 40.28 ± 24.00 n/mm^2^, *P* = 0.044) while the Mecobalamin tablet treatment did not (both *P* > 0.05). Neither treatments led to significantly improved CNFD (both *P* > 0.05). Figure 2e-h were CCM images of the conventional sub-basal nerve plexus pattern around the central cornea patterns before/after the treatment, from which a patient’s pre- and post-treatment CNFL, CNFD and CNBD were measured.

**Table 2:** Efficacy outcomes in patients in the Mecobalamin intramuscular injection group and the oralMecobalamin tablet group (PPS).

Efficacy outcomes		Baseline	8 weeks	*P* value^†^	Changes after 8-week treatment from baseline	*P* value^‡^
IWL (mm/mm^2^)	Injection	17.64 ± 4.83	21.64 ± 3.00	< 0.01*	4.00 ± 3.09	0.002*
	Tablet	22.10 ± 4.74	21.88 ± 4.27	0.816	-0.22 ± 3.92	
CNFL (mm/mm^2^)	Injection	17.60 ± 4.31	20.86 ± 3.46	0.01*	3.26 ± 4.01	0.048*
	Tablet	19.67 ± 3.43	19.75 ± 3.43	0.943	0.08 ± 4.64	
CNFD (n/mm^2^)	Injection	32.64 ± 7.70	38.89 ± 11.18	0.07	6.25 ± 12.22	0.280
	Tablet	35.79 ± 8.32	37.78 ± 6.95	0.404	2.00 ± 9.61	
CNBD (n/mm^2^)	Injection	40.28 ± 24.00	51.76 ± 16.00	0.044*	11.49 ± 20.09	0.090
	Tablet	51.84 ± 20.10	46.98 ± 20.91	0.524	−4.87 ± 30.77	
TCSS (points)	Injection	6.67 ± 2.69	5.27 ± 3.83	0.075	−1.40 ± 2.82	0.228
	Tablet	4.29 ± 1.31	3.76 ± 1.30	0.01*	−0.53 ± 0.72	
SAS	Injection	4.53 ± 2.61	2.40 ± 1.92	< 0.01*	−2.13 ± 1.85	< 0.01*
	Tablet	1.65 ± 1.27	1.47 ± 1.07	0.382	−0.18 ± 0.81	

Data are expressed as means ± standard deviations unless otherwise indicated.

Abbreviations: *CCM* corneal confocal microscopy, *PPS* per protocol set, *IWL* inferior whorl length, *CNFL* corneal nerve fiber length, *CNFD* corneal nerve fiber density, *CNBD* corneal nerve branch density, *TCSS* Toronto clinical neuropathy score, *SAS* survey of autonomic symptoms.

^†^Comparison for efficacy outcomes after 8-week Mecobalamin injection treatment vs oral Mecobalamin tablet treatment.

^‡^Comparison for changes of CCM parameters, TCSS and SAS from baseline for patients receiving Mecobalamin injection treatment vs oral Mecobalamin tablet treatment.

*P value < 0.05.

The Mecobalamin injection treatment led to significantly greater SAS improvement than the tablet treatment (-2.13 ± 1.85 vs -0.18 ± 0.81, *P* < 0.01). The Mecobalamin injection treatment significantly improved patients’ SAS score from baseline (2.40 ± 1.92 vs baseline 4.53 ± 2.61, *P* < 0.01) while the tablet treatment did not (*P* > 0.05) (Table 2). On the other hand, patients in the Mecobalamin tablet group had significantly improved TCSS score at the end of the treatment (3.76 ± 1.30 vs 4.29 ± 1.31 at baseline, *P* = 0.01) while patients in the injection group did not (5.27 ± 3.83 vs 6.67 ± 2.69, *P* = 0.075) (Table 2).

Patients receiving the Mecobalamin injection treatment had significantly improved upper extremity SSR Amp compared to baseline (2.28 ± 1.77 mV vs 1.43 ± 1.20 mV, *P* < 0.05), while patients receiving the tablet treatment did not (1.22 ± 0.99 mV vs 0.97 ± 0.75 mV, *P* > 0.05) (Table 3). Neither treatment led to significant changes in patients’ left sural SNAP, upper extremity SSR Lat, lower extremity SSR Lat or lower extremity SSR Amp (*P* > 0.05) (Table 3).

**Table 3:** SNAP and SSR changes for patients receiving Mecobalamin intramuscular injections vs oral Mecobalamin tablets (PPS).

		Baseline	8 weeks	*P* value^†^	Changes after 8-week treatment from baseline	*P* value^‡^
Left sural SNAP (µV)	Injection	6.79 ± 5.25	10.03 ± 9.76	0.283	2.60 ± 8.35	0.855
	Tablet	8.72 ± 5.14	9.13 ± 6.47	0.769	1.96 ± 8.03	
Upper extremity SSR Lat (ms)	Injection	1391.20 ± 460.18	1292.08 ± 308.33	0.216	−138.08 ± 381.23	0.771
	Tablet	1499.47 ± 458.42	1316.20 ± 175.53	0.124	−99.30 ± 185.22	
Upper extremity SSR Amp (mV)	Injection	1.43 ± 1.20	2.28 ± 1.77	0.027*	0.76 ± 1.08	0.265
	Tablet	0.97 ± 0.75	1.22 ± 0.99	0.735	0.16 ± 1.43	
Lower extremity SSR Lat (ms)	Injection	1655.40 ± 543.36	1704.85 ± 446.87	0.638	−68.54 ± 511.73	0.540
	Tablet	1772.76 ± 363.62	1749.11 ± 288.17	0.872	−227.00 ± 709.10	
Lower extremity SSR Amp (mV)	Injection	0.46 ± 0.37	0.46 ± 0.28	0.861	0.02 ± 0.40	0.763
	Tablet	0.44 ± 0.33	0.41 ± 0.30	0.462	−0.04 ± 0.47	

Data are expressed as means ± standard deviations unless otherwise indicated.

Abbreviations: *SNAP* sural nerve amplitude potential, *SSR* sympathetic skin response, *Lat* latency, *Amp* amplitude.

^†^Comparison for SNAP or SSR after 8-week Mecobalamin injection treatment vs oral Mecobalamin tablet treatment.

^‡^Comparison for changes of SNAP or SSR from baseline for patients receiving Mecobalamin injection treatment vs oral Mecobalamin tablet treatment.

*P value < 0.05.

### Association between CCM parameters changes and SAS change

There was a significant negative correlation between IWL change from baseline and SAS change for patients receiving the Mecobalamin injection treatment (*r* = -0.437 [*P* = 0.012]), indicating correlation between IWL improvement and improvement of patients’ autonomic symptoms, while no significant correlation between SAS change and change(s) of CNFL, CNFD or CNBD (*P* > 0.05) was observed for the injection treatment.

### Adverse events (AEs)

There was no adverse event reported during the study.

## Discussion

In this first study of using CCM to compare efficacy of Mecobalamin intramuscular injections vs oral tablets in treating mild to moderate DPN, we found that patients in the Mecobalamin injection group had significantly improved IWL, CNFL, CNBD and SAS at the end of the treatment compared to baseline while patients in the tablet group did not. Mecobalamin injection treatment also led to significantly greater IWL and CNFL improvements than the tablet treatment. Neither treatment led to significantly improved CNFD. Patients in the Mecobalamin tablet group had significantly improved TCSS score while patients in the injection group did not. Finally patients experience no AEs during the study.

DPN is a disease with extremely complex pathophysiology^1^. The known pathophysiology of DPN includes “increased formation of advanced glycated end products, alterations in protein kinase C pathways, increased polyol pathway activity, decreased nitric oxide/impaired endothelial function, reduced (Na+/K+)-ATPase activity, and homocysteinemia”^25^. Currently, there is no FDA approved treatment for modifying DPN’s underlying pathophysiological changes^5,6^. One possible reason is that these patients’ DPN was often too advanced at this stage to be reversible, therefore, it is uttermost important to detect DPN early especially small fibre damage that occurs early in DPN progression^1,5,9^. On the other hand, it has been reported that IENFD improved in patients with impaired glucose tolerance and neuropathy receiving lifestyle intervention and that CCM parameters improved in patients with type 2 diabetes with improved DPN risk factors^7,11,37^ In addition, a recent study found that patients with Type 1 diabetes and DPN who received SPK showed evident corneal never regeneration 6 months after SPK that continued over 36 months followed by delayed symptomatic and neurological improvement (improved Neuropathy Symptom Profile and peroneal nerve conduction velocity) 36 months after SPK^16^. These studies suggested that the abilities of commonly used therapeutic efficacy endpoints such as neurologic symptoms, electrophysiology and sural nerve fibre morphology might have severe limitation in detecting and defining early therapeutic response to DPN treatments, since electrophysiology and sural nerve fibre morphology only evaluate large nerve fibres, but not small nerve fibres, which are the earliest damaged nerve fibres in DPN^1,5,7,9^. CCM parameters, in this sense, have the potential to be good surrogate endpoints for DPN treatments as these parameters assess Aδ and unmyelinated C fibres enriched corneal nerve fibres, and damage in Aδ and unmyelinated C fibres is responsible for many DPN symptoms^5,7–10^. In addition, CCM parameters can detect pre-clinical DPN and correlate with IENFD^5,7–10,38^. Several additional studies also suggested that CCM could serve as a precise and reliable indication of small nerve fiber damage and regeneration^12–22^, and it has recently been suggested that CCM could be used as a primary end point in clinical studies of DPN disease-modifying treatments^23^. However, it is obvious that more studies are needed to further assess the feasibility of using CCM as a surrogate efficacy biomarker for nerve fibre repair in DPN treatment.

In the current study, we explored the potential of using various CCM parameters as DPN therapeutic endpoints by assessing their changes after 8-week treatment of Mecobalamin intramuscular injections or oral tablets. It has long been reported that Mecobalamin could promote peripheral nerve function/regeneration and improve clinical symptoms in patients with DPN^4,24–28^ by accelerating transmethylation in nerve tissues directly, promoting conversion of homocysteine to methionine, increasing myelination, differentiation and replication of neurons, as well as increasing phospholipids and nucleic acids biosynthesis^25^. It has also been reported that Mecobalamin could correct impaired neural signaling of protein kinase C and ameliorate oxidative stress-induced damage^25^. Mecobalamin is widely used in China for treating peripheral neuropathy, and is also recommended in the Chinese guideline for treating type 2 diabetes^25,28^.

We chose IWL as our primary outcome because it has been reported that IWL reduction occurred earlier than the central cornea nerve fibre reduction and combination of IWL and conventional central corneal nerve parameters such as CNFL and CNFD could improve CCM’s diagnostic performance^30,31^. Our study found that that Mecobalamin injection treatment led to significantly greater IWL and CNFL improvements than the tablet treatment, and that while the injection treatment significantly improved patients’ IWL, CNFL and CNBD, the tablet treatment did not improve any CCM parameters. The greater improvement in corneal nerve morphology associated with the injection treatment was further backed up by the our finding that patients receiving the injection treatment had significantly improved SAS score, while patients receiving the tablet treatment did not, as SAS score is a recognized tool for measuring autonomic dysfunction in early DPN^10,36^. The efficacies of Mecobalamin intramuscular injections and oral tablets have rarely been compared, 2 studies conducted in China suggested that although Mecobalamin intramuscular injection treatment and oral tablet treatment had comparable efficacies in treating DPN after 8 weeks, the injection treatment acted quicker and had significantly greater efficacy than the oral tablet treatment within 4 weeks after treatment initiation^39^^,^^40^. We also found that the injection treatment produced significantly greater improvements in IWL and CNFL than the tablet treatment at week 8, suggesting that IWL and CNFL might be sensitive enough to detect difference in the magnitude of early small nerve fibre repair associated with 2 treatment regimens that could be easily missed by other therapeutic efficacy endpoints. More studies are needed to further explore this point.

One observation of note was that patients receiving the injection treatment had significantly higher baseline TCSS and SAS scores and significantly shorter baseline IWL than patients receiving the tablet treatment (Table 1), suggesting that patients in the injection group had more severe DPN. Patients with more severe DPN are generally expected to recover slower than patients with less severe DPN. Nevertheless, patients receiving the injection treatment showed greater improvement of CCM parameters and SAS, and their IWL improvement correlated with SAS improvement, further suggesting that Mecobalamin injection treatment was more effective in treating DPN than the tablet treatment. On the other hand, we observed that patients receiving the tablet treatment led to significantly improved TCSS treatment (3.76 ± 1.30 vs 4.29 ± 1.31 at baseline, *P* = 0.01) while patients receiving the injection treatment did not (5.27 ± 3.83 vs 6.67 ± 2.69, *P* = 0.075) (Table 3), this result seemed to be inconsistent with our finding of the superior effectiveness of the injection treatment. A closer look revealed that the two treatments led to comparable magnitude of TCSS improvement (−1.40 ± 2.82 [injections] vs −0.53 ± 0.72 [Tablets], *P* = 0.228), however, as patients receiving the injection treatment had a significantly higher baseline TCSS, their post-treatment improvement was close to but not yet statistically significant. As TCSS and SAS evaluate different aspects of DPN (TCSS is “the sum of peripheral symptom score, deep tendon reflex score, and sensory test score”^41^, while SAS assesses patients’ small fibre function by evaluating their autonomic symptoms^10,36^), our finding that the injection treatment led to greater magnitude of SAS improvement and comparable magnitude of TCSS improvement vs the tablet treatment is not that surprising. In addition, we also found that although neither treatment led to significant improvement in left sural SNAP, upper/lower extremity SSR Amp or lower extremity SSR Amp, the injection treatment significantly improved patient’s upper extremity SSR Amp while the tablet treatment did not, further supporting the notion that Mecobalamin injection treatment was more effective in treating DPN than the tablet treatment.

The limitations of the study included the short duration of the treatment, as previous mentioned, due to the COVID-19 pandemic, after the 8-week treatment period and data collection, it became very difficult for some patients to come back for follow-up tests during the originally planned subsequent 24 weeks during which all of the patients took Mecobalamin oral tablets. Therefore, only results from the 8-week treatment are reported. Whether the 8-week Mecobalamin intramuscular injection treatment could maintain its superior treatment efficacy over the 8-week oral tablet treatment by continuing the treatment in the form of oral tablets could not be answered by this study. As oral tablets are much easier to administer, whether the improved treatment efficacy achieved by the injection could be maintained by subsequent oral tablet treatment could be important in determining patient compliance. Secondly, as a preliminary study, this study had a modest sample size. Since there has been no existing data on the effect of Mecobalamin on any CCM parameters, a proper sample size for this study could not be accurately determined and therefore was not calculated. Studies with a larger sample sizes are needed to further confirm our results.

In conclusion, 8-week Mecobalamin intramuscular injection treatment was more effective in treating mild to moderate DPN than oral tablet treatment, and CCM was sensitive enough to detect the difference in the efficacies of the 2 treatments, indicating the usefulness of CCM parameters as surrogate endpoints in DPN therapeutic interventions.

## Data Availability

The data used for this manuscript are available on reasonable request from the corresponding author.
